# M2-AChR Mediates Rapid Antidepressant Effects of Scopolamine Through Activating the mTORC1-BDNF Signaling Pathway in the Medial Prefrontal Cortex

**DOI:** 10.3389/fpsyt.2021.601985

**Published:** 2021-05-17

**Authors:** Shuang Liu, Dandan Shi, Zuoli Sun, Yi He, Jian Yang, Gang Wang

**Affiliations:** ^1^The National Clinical Research Center for Mental Disorder and Beijing Key Laboratory of Mental Disorder, Beijing Anding Hospital, Capital Medical University, Beijing, China; ^2^Advanced Innovation Center for Human Brain Protection, Capital Medical University, Beijing, China

**Keywords:** scopolamine, muscarinic acetylcholine receptor-2, medial prefrontal cortex, mammalian target of rapamycin complex 1, brain-derived neurotrophic factor

## Abstract

**Background:** Scopolamine, a non-selective muscarinic acetylcholine receptor (M1~5-AChR) antagonist, has rapid and robust antidepressant effects in humans and other species. However, which of the five M-AChRs mediates these therapeutic effects has not been fully identified. Several studies implicate M2-AChR as a potential antidepressant target of scopolamine. This study aimed to explore the role of M2-AChR in scopolamine's antidepressant-like effects and determine the underlying mechanisms.

**Methods:** We used the classic novelty suppressed feeding test (NSFT), open field test (OFT) and forced swim test (FST) to observe antidepressant-related behaviors of normal rats, medial prefrontal cortex (mPFC) neuron silenced rats and M2-AChR knockdown rats treated with scopolamine. In a further experiment, the M2 cholinergic receptor antagonist methoctramine (MCT) was injected intracerebroventricularly into normal rats. Levels of mTORC1 and brain-derived neurotrophic factor (BDNF) in the mPFC of animals were analyzed by Western blotting.

**Results:** Consistent with previous studies, mPFC was required for the antidepressant-like effects of scopolamine, and intracerebroventricular injection of MCT into rats could produce similar antidepressant-like effects. Use of AAV-shRNA to knock down M2-AChR in the mPFC resulted in the antidepressant-like effects of scopolamine being blunted. Furthermore, Western blotting demonstrated increased expression of mTORC1 signaling and BDNF in MCT-treated rats.

**Conclusion:** Our results indicate that M2-AChR in the mPFC mediates the antidepressant-like effects of scopolamine by increasing the expression of BDNF and activating the mTORC1 signaling pathway.

## Introduction

Major depressive disorder (MDD) is characterized by anhedonia, loss of motivation and depressive mood. The prevalence of MDD in China is about 3.4%, causing an enormous economic burden on society (1). Drug therapy is the main treatment for MDD. However, the commonly used antidepressant drugs, including selective serotonin reuptake inhibitors (SSRIs) and selective serotonin and norepinephrine reuptake inhibitors (SNRIs), usually take 4–6 weeks to become effective, reducing the medication compliance of patients and having detrimental effects on long-term prognosis (2). Therefore, in recent years there has been an urgent need to research and develop novel, faster-acting antidepressants.

Scopolamine is a non-selective muscarinic acetylcholine receptor (M-AChR) antagonist and has affinity for five M-AChR subtypes, being most selective for M1- and M2-AChR. As an M-AChR antagonist, scopolamine has mainly been used in the treatment of motion sickness, Parkinson's disease, and pregnancy-related vomiting (3). In recent years, however, evidence from clinical trials indicated that scopolamine has a rapid and robust antidepressant effect in both bipolar and unipolar depression. Essentially, a single intravenous injection of low dose (4.0 μg/kg, i.v.) scopolamine can significantly improve depressive symptoms within 3 days and this effect can last for approximately 2 weeks, without serious adverse events. The available clinical evidence shows that it is unlikely to be addictive (4, 5).

It is known that scopolamine exerts rapid antidepressant effects by promoting the release of brain-derived neurotrophic factor (BDNF) and glutamate, activating the mammalian target of rapamycin complex 1 (mTORC1) and enhancing synaptogenesis in the medial prefrontal cortex (mPFC) (6–8). These effects are reported to be mediated by blockade of M1-AChR (8, 9). However, M2-AChR may also be involved in the antidepressant effects of scopolamine (10, 11). Animal studies have shown that scopolamine has no antidepressant-like effects in M1- or M2-AChR knockout mice, but the effects are retained in M3-, M4- or M5-AChR knockout mice (10). Selective M2-AChR antagonists can mimic the antidepressant-like effects of scopolamine (10, 11). Additionally, human studies support the association between M2-AChR and MDD. For example, a polymorphism of the M2-AChR encoding gene, CHRM2, is significantly correlated with development of MDD (12, 13) and MDD patients show reduced binding activity of M2-AChR, but not M1-, M3- and M4-AChR, in the dorsolateral prefrontal cortex (14, 15). Accordingly, we speculate that M2-AChR may be an important antidepressant target of scopolamine. The present study was therefore undertaken to confirm its key role and investigate the underlying mechanisms of M2-AChR in scopolamine's antidepressant-like effects.

## Materials and Methods

### Animals

SPF Sprague Dawley (SD) male rats (total 160), aged 7 weeks and weighing 180–200 g, were pair-housed and maintained in standard conditions with a 12-h light/dark cycle and access to food and water *ad libitum*. They were purchased from Beijing Vitalriver Experimental Animal Center, laboratory animal license number: SCXK (Beijing) 2016-0011. Animal procedures were under the authority of the Animal Ethics Committee of Capital Medical University (ethical permission number: AEEI-2018-017).

### Drugs and Treatments

**Figure 1:**
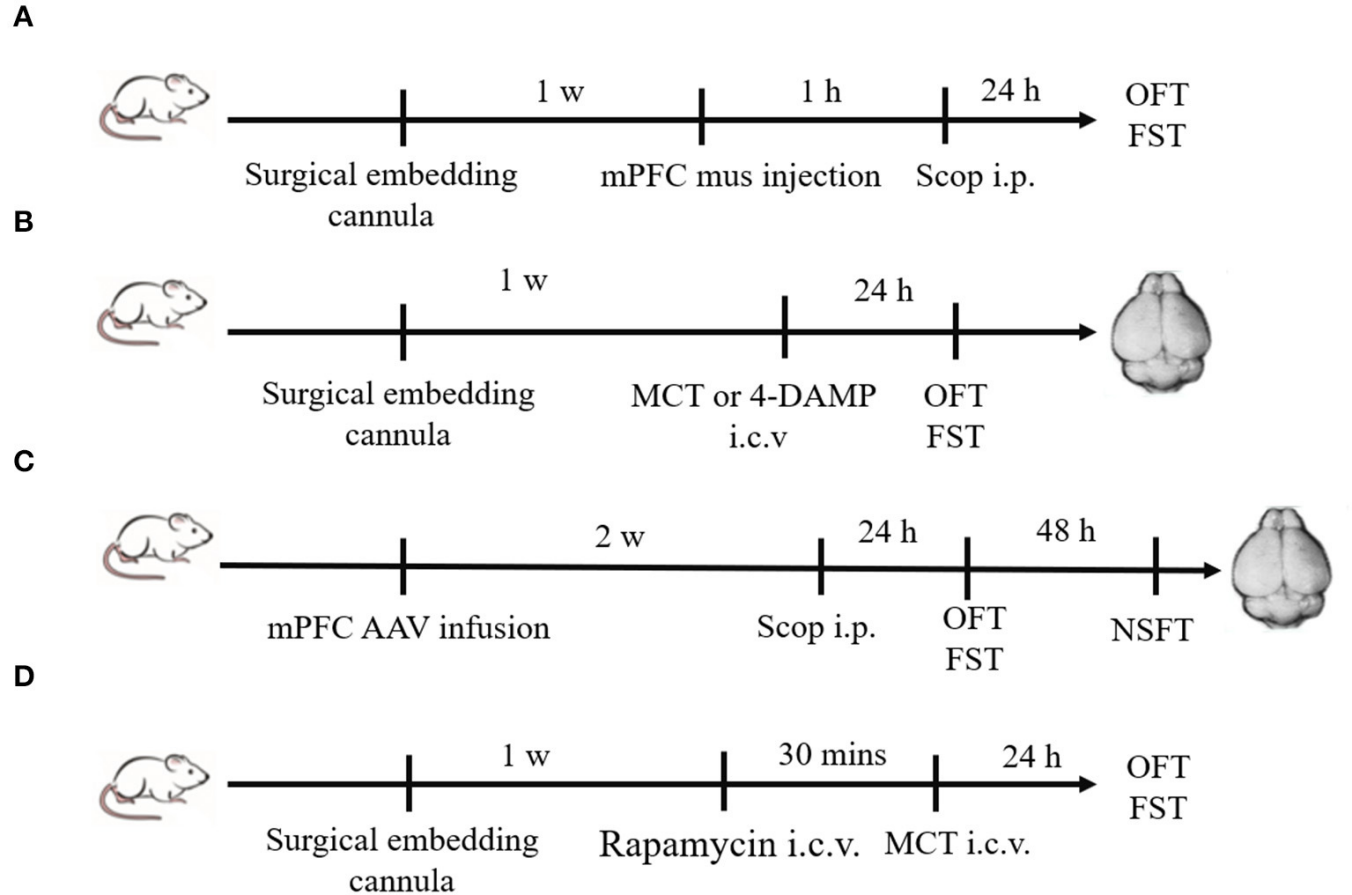
Schematic diagram of experimental procedures. **(A)** Plastic guide cannulas were pre-implanted bilaterally into the mPFC. One week after recovery, muscimol (1.25 μ*g*/2μl) was bilaterally injected through the cannulas 1 h prior to scopolamine administration (i.p.), and behavior tests were conducted 24 h after scopolamine administration. **(B)** Drugs (MCT or 4-DAMP) were bilaterally injected into the lateral ventricle through pre-implanted cannulas, and behavior tests were conducted 24 h later. Then animals were sacrificed, and brains were rapidly removed. **(C)** Two weeks after mPFC AAV infusion, scopolamine was injected (i.p.). Twenty-four hours later, OFT and FST were sequentially conducted. After another 48 h, NSFT were conducted. Then animals were sacrificed, and brains were rapidly removed. **(D)** Rapamycin (0.2 nmol/2 μl) were bilaterally injected into the lateral ventricle through pre-implanted cannulas 30 min prior to MCT administration (i.c.v.). OFT and FST were sequentially conducted 24 h after MCT administration.

Scopolamine (25 μg/kg, Tocris, UK), dissolved in 0.9% saline, was injected intraperitoneally (i.p.). Muscimol (1.25 μg/2 μl, Abcam, USA) dissolved in 0.9% saline was bilaterally injected into the mPFC 1 h prior to scopolamine. M2-AChR antagonist methoctramine (MCT, GlpBio, USA), dissolved in 0.9% saline (0.5 μg/2 μl, 1 μg/2 μl or 2 μg/2 μl), was injected intracerebroventricularly (i.c.v.) (8). M3-AChR antagonist 4-diphenylacetoxy-N-methylpiperidine methiodide (4-DAMP, 100 pmol, Cayman, USA) was dissolved in 0.9% saline (1 μg/2 μl) and injected i.c.v (8). Rapamycin (Solarbio, China), dissolved in dimethyl sulfoxide (0.2 nmol/2 μl), was delivered i.c.v. 30 min prior to MCT injection. Groups of control animals for the above experiments received equal volumes of vehicle alone (0.9% saline or dimethyl sulfoxide). The experimental procedures are illustrated in Figure 1.

### Behavioral Tests

Twenty-four hours after administration of scopolamine, MCT or vehicle, rats were sequentially tested for depression-associated behaviors using the open field test (OFT) followed by forced swim test (FST). Animals were then put back into the cage and 48 h later novelty suppressed feeding tests (NSFT) were conducted.

#### OFT

This test is used to evaluate autonomous behavior and tension levels of rats in a novel environment (16). The rats were individually placed in a coverless plastic square box (100 × 100 × 40 cm). A digital camera covering the entire field was placed above the box. At the beginning of the test, animals were placed in the center of the area and allowed to explore freely for 5 min. The total distance covered was recorded and analyzed by video tracking software (Supermaze, Shanghai XinRuan Information Technology Co., Ltd, China) to estimate effects of drug treatment on locomotor activity.

#### FST

This test is widely used in antidepressant drug research (17). Animals behave with desperation in this test, which is sensitive to most antidepressants. The rats were placed individually into a plexiglass cylinder (barrel height 50 cm, diameter 30 cm, purchased from Wuhan ProBeCare Scientific Inc., China) filled with room temperature water (25 ± 1°C) to a depth of 37 cm, to ensure that animals could not touch the bottom of the container with their hind paws or tails. The time to immobility (only the head of the rat above the water, body floating in the water, limbs slightly moving but not struggling) was recorded within 5 min. The rats were placed in the same environment for 15 min for pre-swimming 24 h before the formal test. EthoVision XT software (version 10, Noldus Information Technology Co., Netherland) was used to analyze the immobility time.

#### NSFT

This test is used to evaluate the anxiety and depression of animals in novel environments (18). Rats were food-deprived for 24 h and placed into a new dimly lit environment (100 × 100 × 40 cm) with food in the center. A digital camera covering the entire field was placed above the box and the latency for feeding (reflecting anxiety-like behavior) in 5 min was recorded. After that, animals were then returned to the home cage immediately and food consumption within 30 min was recorded to eliminate the influence of appetite.

### Adeno-Associated Virus Construction

AAV-CHRM2-shRNA (pAAV-U6-shRNA (Chrm2)-CMV-EGFP-pA) (2.41E + 12vg/ml) purchased from Wuhan BrainVTA Scientific Inc. (Wuhan, China) was injected bilaterally into the mPFC (1 μl in each side). Its empty vector (pAAV-U6-BBSI-shRNA-CMV-EGFP-pA, 2.97E + 12vg/ml) was used as a negative control. The sequence for CHRM2-shRNA was GCCACCTTCAGACTGTCAACA.

### Stereotactic Injection

Rats were anesthetized by 3% sodium pentobarbital solution (30 mg/kg, i.p.) and fixed in the stereotaxic apparatus. For intracerebral injection of drugs, plastic guide cannulas were pre-implanted bilaterally into the infralimbic cortex (IL) of mPFC (AP = 2.8 mm, ML = ±0.6 mm, DV = −3.8 mm) and bilateral lateral ventricle (AP = 0.9 mm, L = ± 1.5 mm, DV = −3.5 mm) (19). One week after recovery, drugs were slowly injected into the target regions at a speed of 0.2 μl/min via a syringe pump (LongerPump Co. LTD, China). Viruses (1 μl on each side) were directly injected bilaterally into the mPFC through a Hamilton syringe using a syringe pump without pre-implantation of guide cannulas. The animals were kept for 2 weeks to allow for virus infection.

### Western Blotting

Animals were sacrificed and brains were rapidly removed and frozen in liquid nitrogen. mPFC was dissected out bilaterally using a brain mold on ice. Homogenates of the dissected tissue were lysed using lysis buffer (Beyotime Biotechnology, China) containing protease inhibitors (Sigma, USA) and phosphorylase inhibitors (Sigma, USA). Electrophoresis was performed on 4–15% Mini-PROTEAN TGX precast gels (Bio-Rad, USA). Primary antibodies used included anti-BDNF (1:500, Abcam, Cat GR3227037-2, USA), -M2-AChR (1:500, Abcam, Cat GR50911-14, USA), -mTORC1 (1:500, Cell Signaling Technology, Cat 2983S, USA), -phospho-mTORC (1:500, Cell Signaling Technology, Cat 5536S, USA) and -β-Actin (1:1,000, Santa Cruz, Cat SC47778, USA). Secondary antibodies used included HRP anti-rabbit antibody (1:5,000, Beyotime, China) and HRP anti-mouse antibody (1:5,000, Beyotime, China). Bands were detected using a chemiluminescence imaging system (Bio-Rad, USA). Image J (NIH, USA) software was used to analyze band densitometry.

### Statistical Analysis

Data are presented as the mean ± standard deviation of the mean (SD). Statistical analysis was conducted using two-tailed Student's *t*-tests (parametric test) and Mann-Whitney *U*-test (non-parametric test) for two-group comparisons. One-way ANOVA or two-way ANOVA was used for three or four-group comparisons, followed by Tukey's multiple comparisons. GraphPad Prism software (version 8.0, GraphPad, USA) was used and differences were considered significant at *p* < 0.05.

## Results

### The mPFC Is Required for Antidepressant-Like Effects of Scopolamine

**Figure 2:**
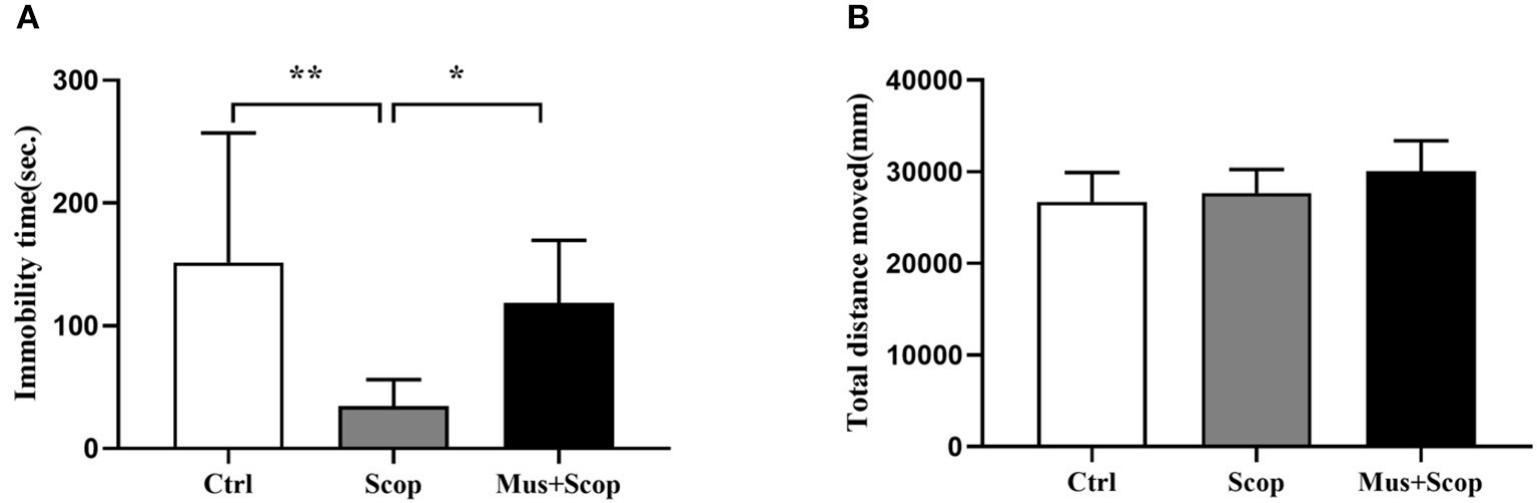
Results for effects of scopolamine on animal behaviors. **(A)** compared with saline group, scopolamine significantly reduces immobility time in the FST in rats, but after silencing of neurons in the mPFC by muscimol, the immobility time of scopolamine is significantly increased compared with scopolamine only. **(B)** total distance among the three groups shows no significant difference. *n* = 10/group; Crtl: control; Scop: scopolamine; Mus: muscimol; *: *p* < 0.05; **: *p* < 0.01.

Previous studies have demonstrated that scopolamine at a dose of 25 μ/kg (i. p.) has rapid and robust antidepressant-like effects on rats, (10, 20–22), and mPFC is a target region of these effects. We replicated these through using a gamma-amino-butyric acid type A (GABA-A) receptor agonist (muscimol) to silence neurons in the mPFC. At the same time, saline and scopolamine were used as two control groups. As shown in Figure 2A (*F* = 6.624, *p* = 0.0053), compared with the saline control group, immobility time in the FST was significantly reduced by scopolamine (*p* = 0.0049), and muscimol significantly increased the immobility time of scopolamine treated rats in the FST (*p* = 0.0412). But there was no significant difference in the locomotor activity in the OFT among these three groups (*p* > 0.05, Figure 2B).

### M2-AChR Mediates the Antidepressant-Like Effects of Scopolamine

**Figure 3:**
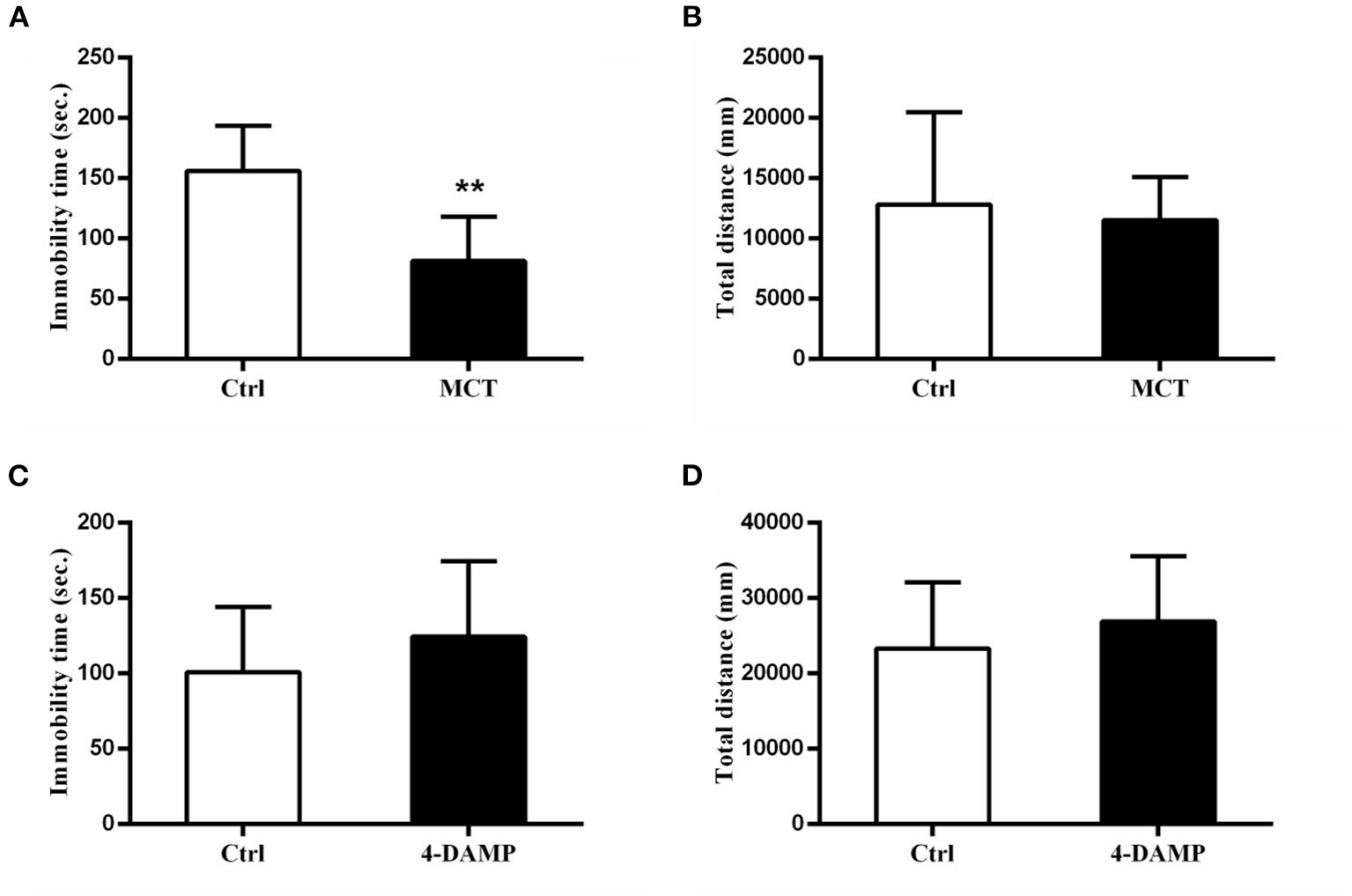
Effects of MCT and 4-DAMP on animal behavior. **(A)** using 1 μg/μl MCT to block M2-AChR in the mPFC significantly reduces the immobility time in the FST. **(B)** total distance between the two groups is not significantly different. **(C)** using 4-DAMP to block M3-AChR cannot significantly reduce the immobility time in the FST. **(D)** total distance between the two groups is not significantly different. *n* = 7–9/group; Crtl: control; **: *p* < 0.01.

MCT, a selective M2 cholinergic receptor antagonist, was used to further explore M2-AChR participation in antidepressant-like effects of scopolamine. Consistent with our previous study (23), we found that intracerebroventricular injection of 1 μg MCT significantly reduced immobility time in the FST (*p* = 0.0081, *t* = 3.906, Figure 3A), while 4-DAMP (100 pmol, i.c.v.), a selective M3-AChR antagonist had no such effect (*p* > 0.05, Figure 3C). In addition, neither significantly affected locomotor activity in the OFT (*p*> 0.05, Figures 3B,D).

**Figure 4:**
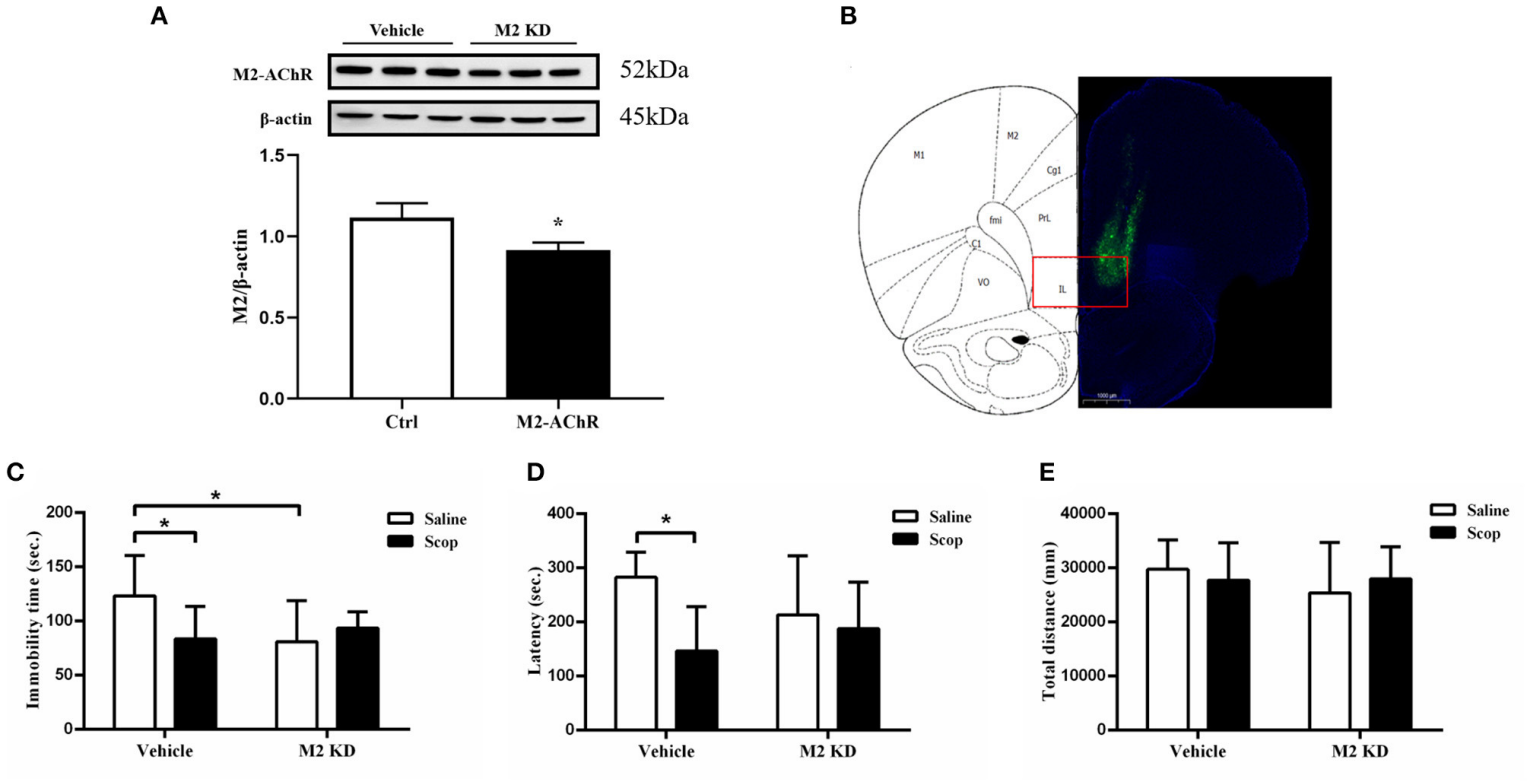
**(A)** Expression of M2-AChR in the M2-KD group was significantly reduced. **(B)** location of frozen section needle path, showing that AAV-CHRM2-shRNA (pAAV-U6-shRNA (Chrm2)-CMV-EGFP-pA) was injected into the mPFC. **(C)** immobility time of the M2-KD-saline group and Ctrl-scop group is significantly reduced. **(D)** latency of the Ctrl + saline group in NSFT is significantly reduced. **(E)** total distance between these groups shows no significant difference. *n* = 7–9/group; Crtl: control; Scop: scopolamine; *: *p* < 0.05.

To further investigate the role of M2-AChR in the antidepressant effects of scopolamine, we injected an AAV vector expressing CHRM2-shRNA (AAV-CHRM2-shRNA) into the mPFC bilaterally, which produced an ~35% reduction in M2-AChR protein levels in the mPFC (*p* = 0.0253, *t* = 3.484, Figures 4A,B). Compared with empty vector + saline group, M2-AChR knockdown (KD) induced a significant decrease of immobility time in the FST (*F* = 5.261, *p* = 0.0317, Figure 4C). However, scopolamine could no longer reduce the immobility time in M2-AChR KD rats (Figure 4C) and M2-AChR KD rats showed a similar effect of scopolamine (*p* = 0.0351, Figure 4C). Similar results were obtained in the NSFT, scopolamine significantly reduced the latency to feeding in the empty vector group (*F* = 7.077, *p* = 0.0309, Figure 4D), while M2-AChR KD blocked this effect (Figure 4D). Home cage food consumptions among the four groups were not statistically different. There was no statistical difference in total distance in the OFT among these groups (*p* > 0.05, Figure 4E). Taken together, these findings indicate that M2-AChR is required for the antidepressant-like effects of scopolamine.

### mTORC1 Signaling Is Essential for the Antidepressant Effects of MCT

**Figure 5:**
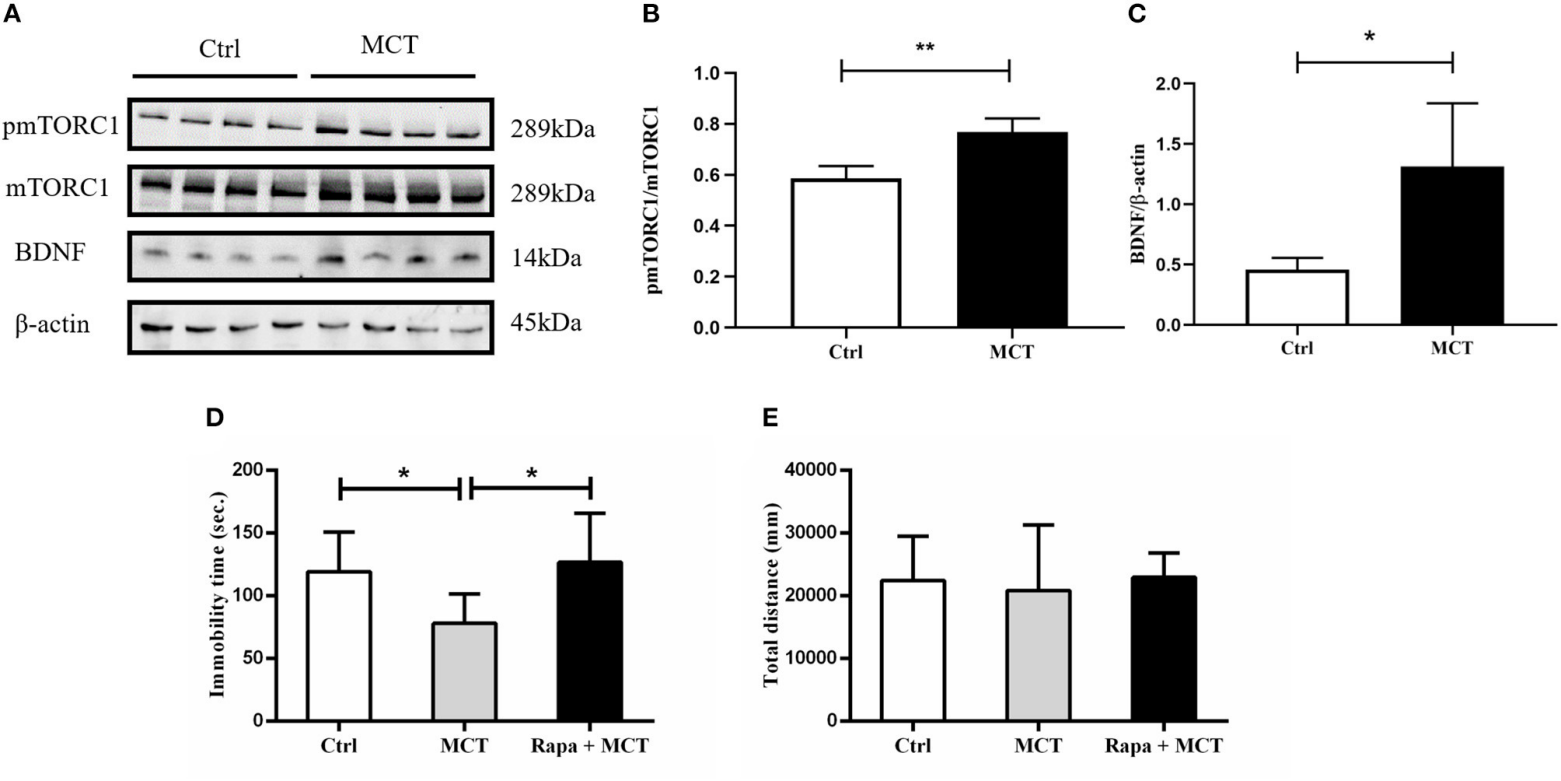
Western blot results from the mPFC and the effect of rapamycin on animal behavior. **(A)** Western blot images of the control group and MCT group; **(B)** MCT significantly increases the ratio of p-mTORC1/mTORC1; **(C)** MCT significantly increases the expression of BDNF; **(D)** compared to the control group, MCT significantly reduces the immobility time in the FST; **(E)** total distance in the OFT is not significantly different from controls, the MCT group and rapamycin + MCT group. *n* = 4/group; Crtl: control; Scop: scopolamine; Rapa: rapamycin; *: *p* < 0.05; ** *p* < 0.01.

It has been demonstrated that scopolamine exerts rapid antidepressant-like effects through activation of the mTORC1 signaling pathway. Here, we observed similar effects of MCT on mTORC1 signaling in the mPFC (*p* = 0.0023, *t* = 5.053, Figures 5A,B). In addition, MCT significantly increased the expression of BDNF in the mPFC (*p* = 0.0182, *t* = 3.218, Figures 5A,C). MCT induced a significant decrease of immobility time in the FST (*F* = 5.371, *p* = 0.0131; *p* = 0.0447, Figure 5D). However, after pretreatment with the mTORC1 inhibitor rapamycin (0.2 nmol/2 μl, i.c.v,), the antidepressant-like effect of MCT in the FST was completely inhibited (*p* = 0.0163, Figure 5D). These results suggest that the BDNF/mTORC1 signaling pathway is critical to the antidepressant-like effects of MCT.

## Discussion

Both human and animal studies have demonstrated that scopolamine has rapid and robust antidepressant activities. However, as a non-selective M-AChR antagonist, scopolamine acts at all five M-AChRs (M1-M5) with equal potency, and it has not been fully determined which one of them mediates these therapeutic effects. In the present study, we found that M2-AChR in the mPFC is required for the antidepressant-like effects of scopolamine, and enhancement of the BDNF/mTORC1 signaling pathway may be the downstream mechanism antagonizing M2-AChR.

Dysfunction of the mPFC has been linked to the cognitive and emotional deficits in depression (24). Herein, our results reveal that silencing neurons in the mPFC could abolish the antidepressant-like effects of scopolamine (Figure 2), identifying the mPFC as a critical brain region for the behavioral effects of scopolamine. This is consistent with the finding by Voleti et al. that scopolamine administration rapidly increased neuronal activity in the mPFC (20). Also, ketamine, as a non-competitive glutamate N-methyl-D-aspartate (NMDA) receptor antagonist, has been shown to rapidly relieve depression symptoms through enhancement of α-amino-3-hydroxy-5-methyl-4-isoxazole propionic acid receptor (AMPA) receptor signaling in the mPFC (25). Moreover, both scopolamine and ketamine have been demonstrated to rapidly stimulate glutamate/GABA-glutamine cycling in the mPFC, which is critical in initiating rapid-acting antidepressant activity (25, 26). In addition to the mPFC, the hippocampus may also be involved in the antidepressant like effects of scopolamine. Several rodent studies have shown that scopolamine induces PKA-dependent AMPA receptor potentiation and increased expression of BDNF and the neuropeptide VGF in the hippocampus (11, 22). Other brain regions, such as the lateral habenula, nucleus accumbens and others, are also reactive to scopolamine administration (20). Therefore, it is likely that scopolamine acts on emotion-related circuits composed by multiple brain regions to exert its antidepressant effects, and this complexity necessitates further study.

In relation to the specific antidepressant target of scopolamine, most of the previous studies focus on the M1-AChR. In the brain, M1-AChR is mainly located in the hippocampus, striatum medium spiny neurons, cortical pyramidal neurons, amygdala, and thalamus postsynaptic. It is expressed in glutamatergic pyramidal neurons and GABA interneurons with increased excitability and its physiological functions include learning, memory, inflammatory cytokine production, etc. (27, 28). A recent study showed that M1-AChR in mPFC somatostatin-GABA interneurons is antagonized by scopolamine, leading to disinhibition of pyramidal glutamate neurons, increased glutamate release and enhanced numbers and function of synapses. A study of the M1- and M3-AChR antagonist penehyclidine hydrochloride (PHC) also gave similar results: PHC could improve depressive-like behaviors in depression model mice and increased BDNF expression could be observed in the hippocampus (29). By contrast, specifically knocking down the M1-AChR of SST-GABA interneurons weakened the rapid antidepressant-like effect of scopolamine (9). These results show that M1-AChR is a main target of scopolamine's rapid antidepressant-like effects. In our study, we found that intracerebroventricular injection of MCT produced similar antidepressant-like effects to scopolamine in the FST (Figure 3) and knocking down M2-AChR in the mPFC blocked scopolamine's antidepressant-like effects in the FST (Figure 4). This is consistent with the results of a previous study that M2-AChR knockout (KO) mice had a blunted response to scopolamine in the FST and the antagonist SCH226206, which has selectivity for M2-AChR over M1-AChR, was effective in the FST but the effect was negated in M2-AChR KO mice (10). M2-AChR is mainly located in the basal forebrain, thalamus, brainstem, heart and exocrine glands (27), and scopolamine therapy may have some dry mouth and cardiovascular-related adverse effects. However, no trials have been halted due to serious adverse events (21, 30).

M1-AChR studies have found that scopolamine can stimulate glutamate transmission or glutamate burst, resulting in a long-term potentiation–like synaptogenic effect (8). Reports of the downstream mechanisms of M2-AChR antidepressant-like effects, however, are rare. Here, we found that MCT activated mTORC1 signaling and increased BDNF expression in the mPFC and inhibiting mTORC1 with rapamycin could block antidepressant-like effects of MCT (Figure 5). The mTORC1 pathway is implicated in activity-dependent synaptic plasticity, especially in neuronal dendrites and spines (31). Dong et al. reported that scopolamine blocks M2-AChR, activating the PKA signaling pathway, which results in mTORC1 pathway activation and synaptic plasticity enhancement (11). The rapid antidepressant effect of ketamine also relies on the rapid activation of mTORC1 signaling, resulting in activation of synapse-associated proteins, such as extracellular signal–regulated kinase (ERK), protein kinase B (PKB/Akt) and phosphorylated 70S6 kinase (p70S6K), increasing in spine number in the PFC and release of BDNF in the amygdala (32, 33). Increased expression of mTORC1 related proteins including phosphorylated ERK 1/2, phosphorylated Akt, p70S6K and others, is required for synaptic and antidepressant effects (6). Animal studies have similar conclusions: both the phosphorylation of mTORC1 and its downstream protein p70S6K are significantly reduced in depressive model animals (34). Protein kinase is well-known to be involved in glutamic synaptic enhancement and synaptic plasticity and patients with depression have altered AMPA receptor expression, which is closely related to the pathophysiology of depression (35). Nonetheless our study has the limitation that its related mechanisms were not explored. As mentioned above, scopolamine could stimulate BDNF and VGF release in the hippocampus and PFC and after using verapamil to block L-type voltage-dependent calcium channels (L-VDCC), antidepressant-like behavior and upregulation of BDNF and VGF were blunted (22). BDNF is an important neurotransmitter involved in regulation of emotions. BDNF expression in depressive animal models is decreased (36) and animal work has also shown that acute administration of ketamine improves BDNF and mTOR levels in the hippocampus, so the mTORC1-BDNF signaling pathway is probably the target of the rapid antidepressant effect (37). Together, these data indicate that regulating BDNF expression and mTORC1 signaling in the PFC and hippocampus may be involved in the rapid antidepressant effects of scopolamine. Further research is underway and answering these remaining questions will have a profound impact on the development of novel rapid-acting antidepressants.

As mentioned, there are limitations in the current study. First, we did not directly examine whether scopolamine activates mTORC1 and increases BDNF expression via M2-AChRs in the mPFC. Secondly, our findings were not validated in animal models of depression (e.g., the chronic unpredictable stress model). Lastly, the current study used only male rats, so our findings may not be generalizable to female rats.

## Conclusion

Our current data from rats suggest that the antidepressant-like behaviors of scopolamine may depend on blockade of M2-AChR and activation of the mTORC1-BDNF signaling pathway in the mPFC. In addition, our findings indicate that intracerebroventricular injection of a defined dose of the M2-AChR antagonist MCT has similar behavioral effects and responses at a molecular level.

## Data Availability Statement

The raw data supporting the conclusions of this article will be made available by the authors, without undue reservation.

## Ethics Statement

The animal study was reviewed and approved by Animal Ethics Committee of Capital Medical University.

## Author Contributions

All authors listed have made a substantial, direct and intellectual contribution to the work, and approved it for publication.

## Conflict of Interest

The authors declare that the research was conducted in the absence of any commercial or financial relationships that could be construed as a potential conflict of interest.
