# Supplementary figures and images for: M2-AChR Mediates Rapid Antidepressant Effects of Scopolamine Through Activating the mTORC1-BDNF Signaling Pathway in the Medial Prefrontal Cortex
Source: Front Psychiatry. 2021 May 17;12:601985. doi: 10.3389/fpsyt.2021.601985 (PMC8176437; doi:10.3389/fpsyt.2021.601985)

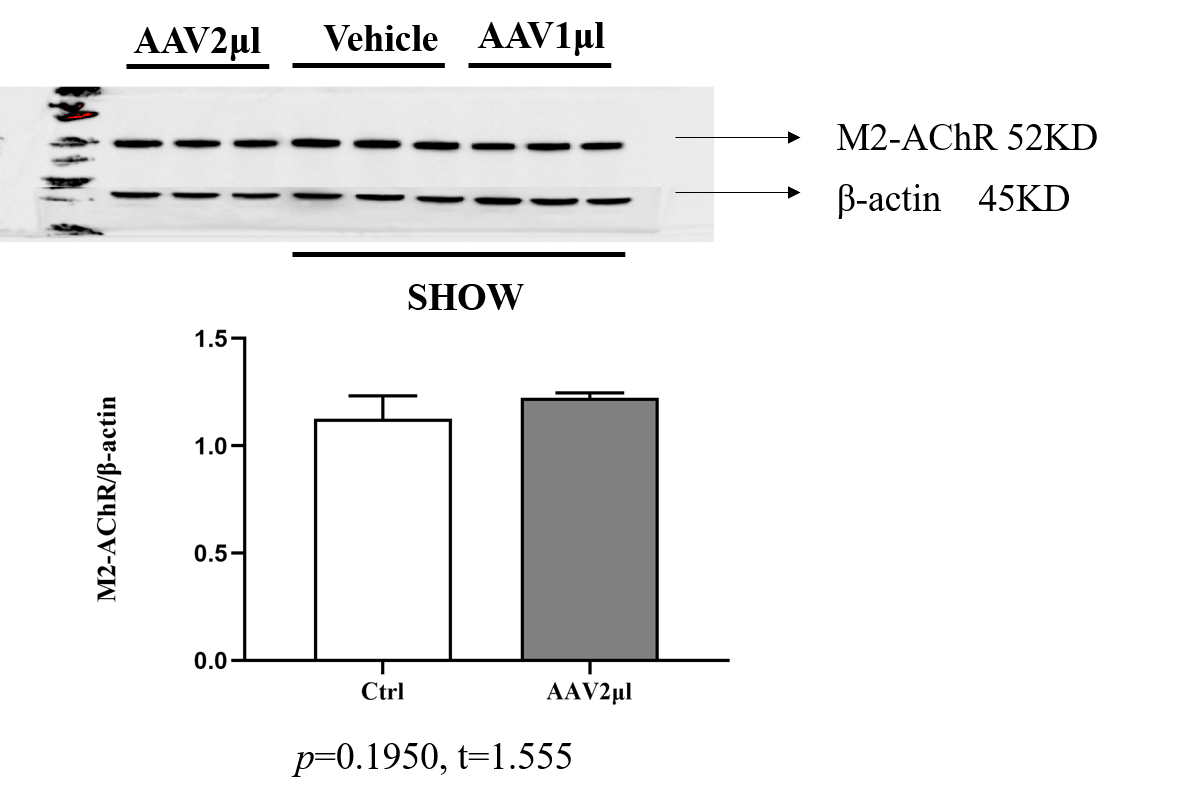

Supplement: Supplementary file 1 [file Data_Sheet_1.ZIP › original images/Figure 4/AAV2a╠l.tif]

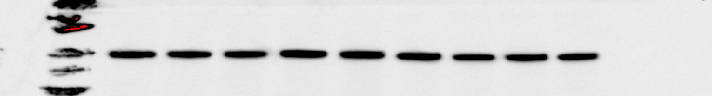

Supplement: Supplementary file 1 [file Data_Sheet_1.ZIP › original images/Figure 4/M2-AChR.tif]

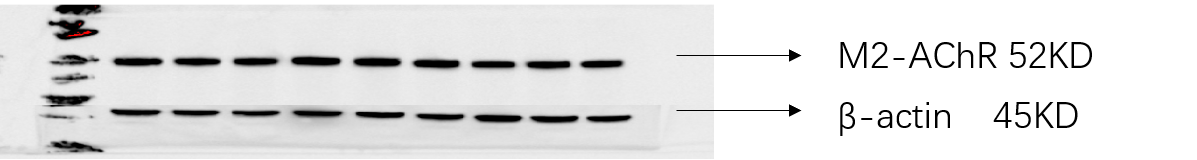

Supplement: Supplementary file 1 [file Data_Sheet_1.ZIP › original images/Figure 4/M2-AChR_actin.tif]

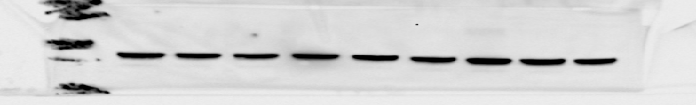

Supplement: Supplementary file 1 [file Data_Sheet_1.ZIP › original images/Figure 4/a┬-actin.tif]

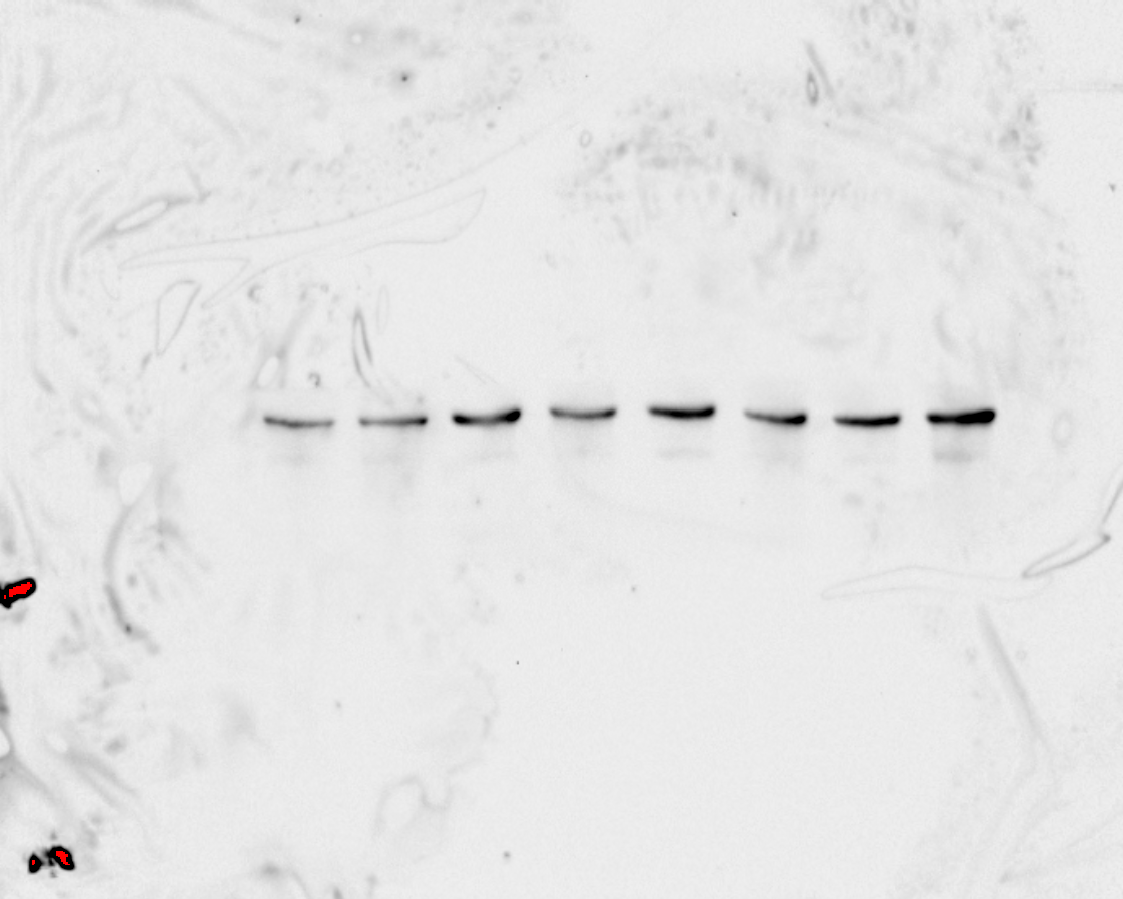

Supplement: Supplementary file 1 [file Data_Sheet_1.ZIP › original images/Figure 5/BDNF/12-11 BDNF-actin.tif]

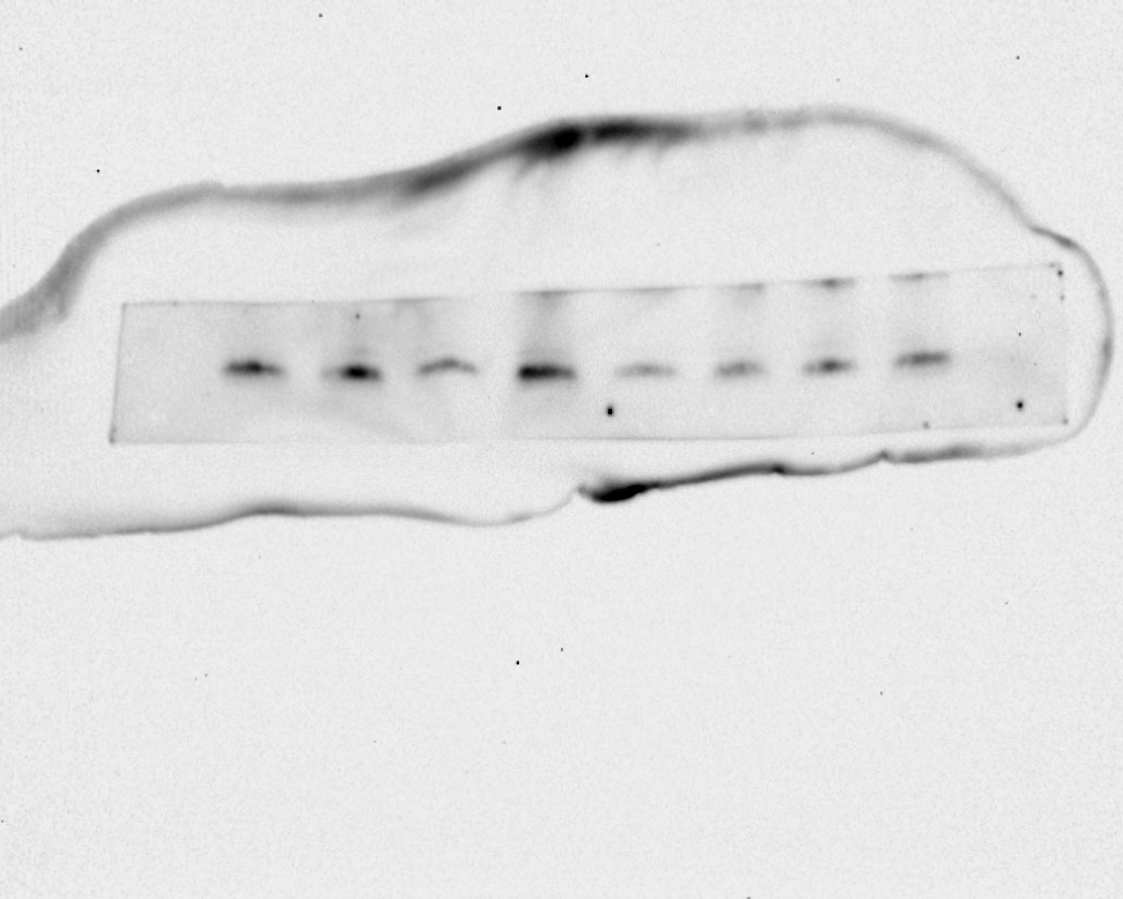

Supplement: Supplementary file 1 [file Data_Sheet_1.ZIP › original images/Figure 5/BDNF/12-11BDNF.tif]

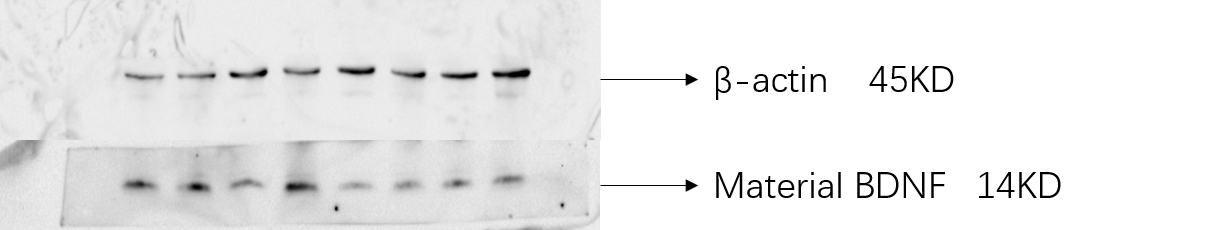

Supplement: Supplementary file 1 [file Data_Sheet_1.ZIP › original images/Figure 5/BDNF/12.11BDNF.tif]

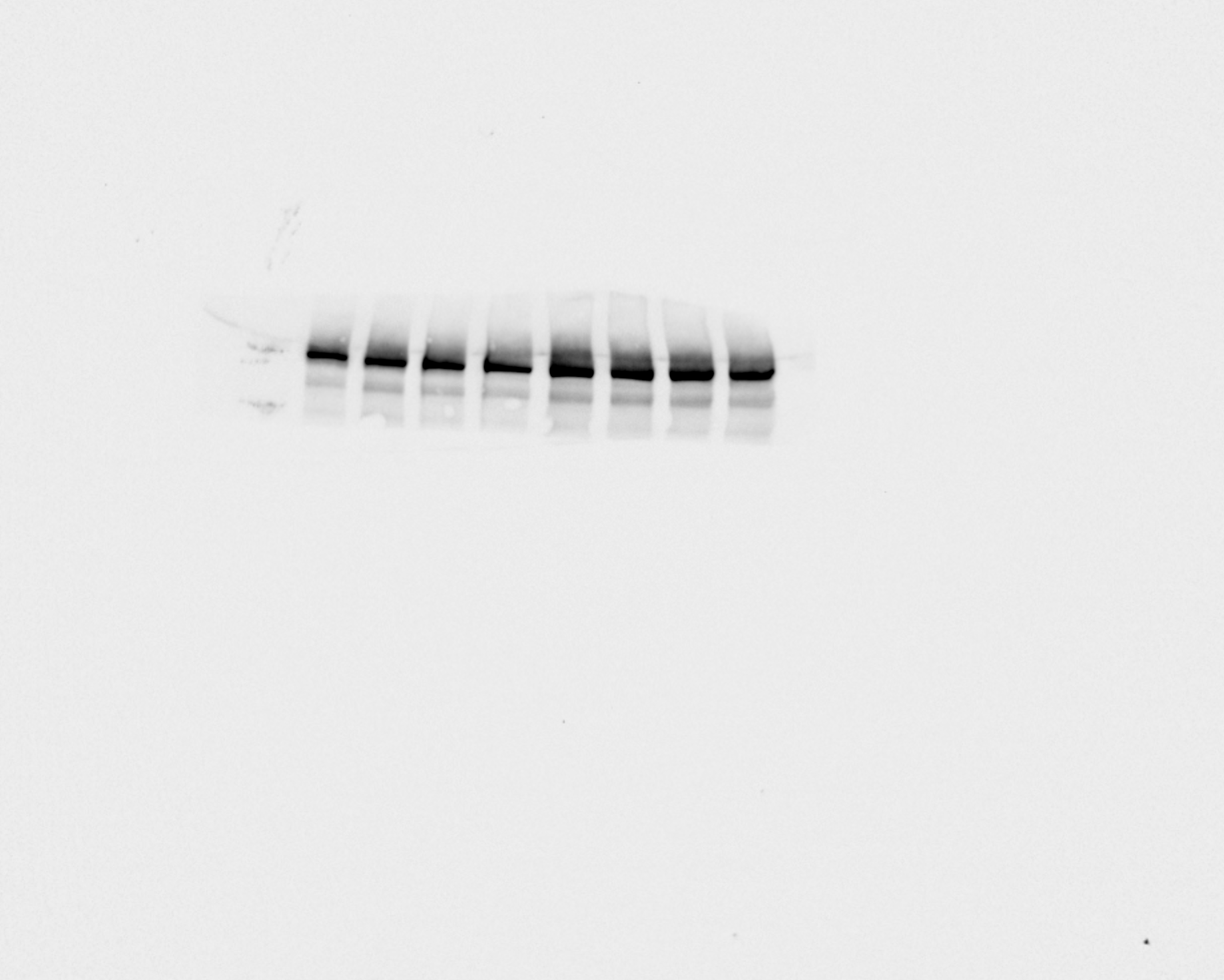

Supplement: Supplementary file 1 [file Data_Sheet_1.ZIP › original images/Figure 5/mTOR/1.30MCT_mtor.tif]

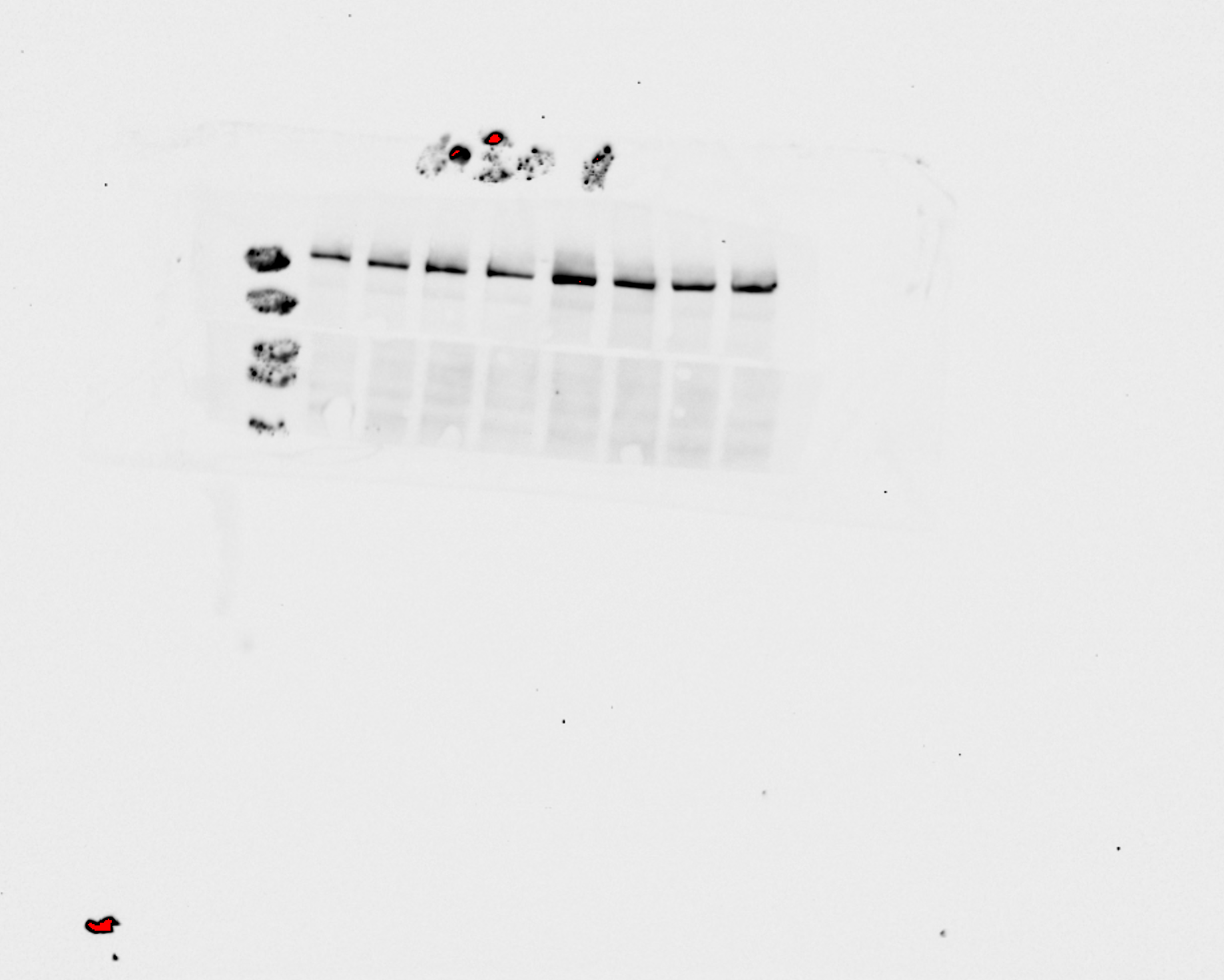

Supplement: Supplementary file 1 [file Data_Sheet_1.ZIP › original images/Figure 5/mTOR/1.30MCT_pmtor.tif]
